# Changes in serum lipids with the onset and progression of Diabetic Retinopathy in Type-II Diabetes Mellitus

**DOI:** 10.12669/pjms.39.1.6265

**Published:** 2023

**Authors:** Mehnaz Nuruddin Gitay, Arisha Sohail, Yasmeen Arzoo, Muhammad Ali Shakir

**Affiliations:** 1 Dr. Mehnaz Nuruddin Gitay, Ph.D. Assistant Professor, Department of Biochemistry, Dow University of Health Science, Karachi, Pakistan; 2 Dr. Arisha Sohail, M.B.B.S, Ph.D. Lecturer, Department of Biochemistry, Dow University of Health Science, Karachi, Pakistan; 3 Yasmeen Arzoo, Institute of Basic Medical Sciences, Dow University of Health Science, Karachi, Pakistan; 4 Muhammad Ali Shakir, M.B.B.S, M.Phil. Professor and Head of Department of Biochemistry, Karachi Medical and Dental College, Karachi, Pakistan

**Keywords:** Diabetic Retinopathy, Dyslipidemia, Type-II Diabetes Mellitus

## Abstract

**Objective:**

To investigate the role of serum lipids in the onset and progression of Diabetic Retinopathy (DR) in Type-II diabetes (T2DM) individuals.

**Methods:**

This cross-sectional study was conducted at the National Institute of Diabetes and Endocrinology (NIDE), Dow University of Health Sciences (DUHS) from March to May 2019. After signing the informed consent, healthy controls (n=30), T2DM patients (diabetic n=30), proliferative (PDR n=30) and non-proliferative (NPDR n= 30) of age 18 to 65 years were selected by convenient sampling. Background information was gathered through interviews and the fundoscopy was done. Fasting five ml venous blood samples were collected and analysed for triglycerides (TGs), cholesterol, HDL, LDL, VLDL and the HbA1c using commercially available assays. The SPSS, version 24.0, was used for data analysis.

**Results:**

The HbA1c level was high in the diabetes, NPDR and PDR groups than control (p<0.05). The serum TGs and cholesterol were raised while the HDL was low in the diabetes group than in control (p<0.05). The cholesterol and LDL were high in the diabetes group compared to NPDR and PDR groups (p<0.05). The cholesterol and VLDL showed a positive moderately strong correlation with HbA1c in the PDR group (p<0.05).

**Conclusion:**

The serum lipid levels vary with the HbA1c levels and greater degree of derangement is observed with increasing mean HbA1C independent of diabetic retinopathy. For this reason, strict control of HbA1c and serum lipid level by lifestyle and/or pharmacologic intervention is recommended in diabetes with or without retinopathy.

## INTRODUCTION

Diabetic retinopathy (DR) occurs due to microvascular complications affecting the eye in Diabetes Mellitus. It is the leading cause of blindness among people aged 55 or older worldwide.^1^ Diabetic retinopathy may be non-proliferative diabetic retinopathy (NPDR) and proliferative diabetic retinopathy (PDR). The development of new blood vessels (retinal neovascularization) forms the basis of its classification The initiating factors of DR has led to the consideration of poor glucose control of major importance and the events originating from hyperglycemia lead to onset and progression of diabetic retinopathy.^2^ The meta-analysis of 261 studies upto 2014 found an increasing trend of blindness and impaired vision due to diabetic eye complications worldwide. DR prevalence raised by 7.7% for blindness and by 28.6% for low vision and is predicted for increase.^3^ The meta-analysis study in Asian Type-II diabetics found the prevalence of DR, PDR, and NPDR was 28%, 6%, and 27% respectively and observed that NPDR is more prevalent than PDR in them.^4^

Pakistan ranks fifth in diabetes burden which is expected to be twice by 2025.^5^ The prevalence of DR in Pakistan was estimated to be 28.78% in all diabetics but observed with inconsistent frequency of DR and PDR.^6^ Rizwan et al., reported low awareness and outcomes of diabetic retinopathy in diabetics in Pakistan^7^ Early detection, strict vigilance of blood glucose, blood pressure, and lipids may decrease the risk of developing DR. Dyslipidemia in Diabetes Type-I and II is strongly associated with the onset and progression of DR Dyslipidemia plays role in early events of DR development^8^ but the link between peripheral lipids and retinal microvascular pathology in T2DM is still not clear. In view of this, the present study aims to investigate the role of serum lipids in the onset and progression of Diabetic Retinopathy in Type-II diabetic individuals.

## METHODS

This cross-sectional study was conducted at the National Institute of Diabetes and Endocrinology (NIDE), Dow University of Health Sciences (DUHS) from March to May 2019 after the ethics committee approval of IRB-1212/DUHS/Approval/2019. After signing the informed consent Form, Type-II diabetic patients without (n=30) and with diabetic retinopathy (Proliferative n=30, non-proliferative n= 30) aged 18 to 65 years were selected by convenient sampling and compared with healthy controls (n=30). Information regarding age, gender, smoking and alcohol consumption, family history and duration of DM, drug history of insulin and oral hypoglycemic agents, and hypertension was gathered through interview.

The fundoscopy of selected patients was performed, and the presence and findings of diabetic retinopathy were noted. After fasting of eight hours or more five ml venous blood samples were collected by a phlebotomist under standard conditions. The fasting triglycerides (TGs), cholesterol, HDL, LDL, VLDL and the HbA1c levels were performed at the Dow Lab Diagnostic Reference & Research Laboratory using commercially available assays. The Statistical Program for Social Sciences (SPSS, version 24.0, IBM) for Windows was used for statistical analysis. The p values p<0.05 were considered significant.

## RESULTS

**Fig.1:**
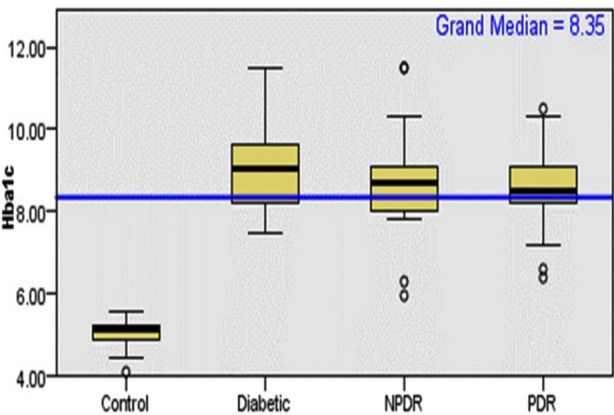
Distribution of serum glycated haemoglobin (HbA1c) across the study subjects (n=120).

The mean age of diabetes, NPDR and PDR groups were 55.93 years. The average fasting serum triglycerides (TGs), cholesterol, HDL, LDL, and HbA1c levels of study subjects is shownin Table-I. The highest HbA1c level in the diabetic group was 9.0 mg/dL in comparison of control, NPDR and PDR groups.Fig-1.

**Table-I:** General characteristics, serum glycated haemoglobin (HbA1c) and lipid profile of study subjects (n=90).

Variables	Mean ± S.D
Age (years)	55.93 ± 7.08
HbA1c (mg/dL)	7.87 ± 1.88
TGs (mg/dL)	125.94±16.97
Cholesterol (mg/dL)	177.43±19.29
LDL (mg/dL)	95.44±13.22
HDL (mg/dL)	45.90±7.11
Non-HDL Cholesterol (mg/dL)	106.76±14.13
VLDL (mg/dL)	25.11±3.96

The pairwise comparisons of the four groups across multiple tests (adjusted for Bonferroni correction) as shown in Table-II. Compared with the control group, the serum fasting HbA1c was significantly increased in diabetes, NPDR and PDR groups (p<0.05). Compared with the control group, serum TG and cholesterol levels were increased, while HDL levels were significantly decreased (p<0.005). Cholesterol and LDL levels were significantly higher in the diabetes group compared to the NPDR and PDR groups (p<0.005).

**Table-II:** Pairwise comparisons of serum glycated haemoglobin (HbA1C) and lipid concentrations in study subjects (n=120).

Groups	Serum lipid biomarkers						

	HbA1c (mg/dL)	TGs (mg/dL)	Cholesterol (mg/dL)	LDL (mg/dL)	HDL (mg/dL)	Non-HDL Cholesterol (mg/dL)	VLDL (mg/dL)
Control^a^	5.0 (1.49)	119.0 (103)	165.0 (62)	94.0 (43.0)	48.0 (21.0)	97.0 (60)	26.0 (12)
Diabetic^b^	9.0*a (4)	132.0**a (61)	196.0**a (93)	103.5 (66)	41.0**a (19)	102.0 (44)	27.0 (17)
NPDR^c^	8.7*a (5.5)	122.5 (61)	174.0**b (59)	93.0^**b^ (26)	47.5 (20)	106.0 (41)	26.0 (13)
PDR^d^	8.5*a (4.1)	134.0**a (42)	173.0**b (47)	91.0**b (36)	47.0 (16)	108.0*a (43)	24.5 (13)
p-value	<0.05*	<0.005**	<0.005**	<0.005**	<0.005**	<0.05*	>0.05

All values showed as median values and range,

* P<0·05,

** P<0.005;

Kruskal-Walis test was used to compare the significance of difference among the groups.

NPDR=Non-proliferative diabetic retinopathy; PDR= Proliferative diabetic retinopathy.

The correlations of serum HbA1c and lipid biomarkers in each group as shown in Table-III. The cholesterol and VLDL are positively and significantly correlated with HbA1c in PDR group.

**Table-III:** Correlations between serum glycated haemoglobin (HbA1c) and lipid concentrations in study subjects (n=120).

Serum lipid biomarkers						

Groups	TGs mg/dL	Cholesterol mg/dL	LDL mg/dL	HDL mg/dL	Non-HDL Cholesterol mg/dL	VLDL mg/dL
Control (n=30)	0.09	0.32	0.13	0.03	-0.23	0.19
Diabetic (n=30)	-0.01	-0.28	-0.14	-0.30	-0.24	-0.09
NPDR (n=30)	0.07	-0.15	0.17	0.01	-0.15	0.23
PDR (n=30)	0.11	0.38*	-0.09	-0.19	0.28	0.43*

* P<0·05; NPDR=non-proliferative diabetic retinopathy; PDR= Proliferative diabetic retinopathy.

## DISCUSSION

In the present study, the fasting serum TGs, cholesterol, LDL and HbA1c levels are significantly raised in diabetic and retinopathy groups except the HDL level which was low in the diabetes group as compared to the control group. Das and Banik^9^ reported the similar dyslipidemic pattern prevalent in diabetic patients in southern Bangladesh.

The significantly high HbA1c, cholesterol and LDL levels in diabetes group as compared to the NPDR and PDR groups shows increased risk of diabetic complications and indicates the progression towards the development of DR as these changes stimulated by lipids are key to the DR pathology^10^,^11^. There is a strong clinical evidence that dyslipidemia predicts events and development of retinopathy in diabetic patients in absence of DR. The literature suggests combined mechanisms play key role in progression of DR in the presence of abnormal lipid and glucose metabolism^12^. Literature shows much evidence of association between increased HbA1c levels and DR incidence. The reason of lower HbA1c levels in NPDR and PDR groups could be better glycemic control which predicts better prognosis for DR^13^. A comparative study in Pakistani population reported significantly deranged serum lipid subfractions in DR group as compared to diabetics without DR but the former group showed significantly higher HbA1C than the diabetics.^14^ However, there is unclear evidence of differences in serum TG, cholesterol, and HDL levels between patients with DR and without DR.^13^ There is evidence suggesting link between dyslipidemia in diabetes and insulin effects on liver apoprotein production, regulation of lipoprotein lipase, actions of cholesteryl ester transfer protein (CETP), and peripheral actions of insulin on adipose and muscle tissue.^15^ In present study the decreased cholesterol and LDL levels in DR group than in the diabetes may be attributed to the better glycemic control and dyslipidemia management after diagnosis of retinopathy. This finding is supported by Amin et al^14^ who found strong positive correlation between HbA1c and severity of DR in Pakistani population and Li Na et al.^16^ observed higher risk of DR is associated with lower level of diabetic self-management in Chinese population.

The decreased HDL levels in diabetes group of present study coincide with the raised TGs level indicating a high free fatty acid flux in response to active lipases. This finding is further supported by the significant positive correlation of mean HbA1c with VLDL in the PDR group. However, the literature lacks evidence of TGs as an independent risk factor of DR^17^ but increase in triglycerides adds synergistic effect for PDR progression, when all lipid types are raised. This study moderately correlates TC, VLDL levels and mean HbA1c in the PDR group. Similar results were reported in TDM2 patients in Chinese population.^18^,^19^ The present study did not correlate the mean HbA1c and serum TC, TGs and LDL in diabetic and NPDR groups. Hence, the role of cholesterol in progression of DR also remains unclear.^20^,^21^ The differences in ethnicity, lifestyle, diet and study methods contribute to varying findings of serum lipids in diabetic and retinopathy groups in different studies. This study suggests that although correlation between HbA1c and lipid subfractions was non-significant but the best measures to control HbA1C may improve the serum lipid level and hence decrease the risk of diabetic dyslipidemia complications.

### Strength and Limitation of study:

The number of participants in each of the control, diabetes, NPDR, and PDR groups was the same. However, due to the cross-sectional design of the study, the temporal relationship between dyslipidemia and DR could not be determined.

## CONCLUSION

The serum lipid levels vary with the HbA1c levels and greater degree of derangement is observed with increasing mean HbA1c independent of diabetic retinopathy. This also raises the important question whether similar serum lipid changes are likely to occur in other diabetic complications. For this reason, strict control of HbA1c and serum lipid level by lifestyle and/or pharmacologic intervention is recommended in diabetes with or without retinopathy.
